# The attitudes to inclusion and Teacher Efficacy for Inclusive Practices scales: psychometric properties across Swiss in-service, pre-service, and special education teachers

**DOI:** 10.3389/fpsyg.2025.1531782

**Published:** 2025-11-19

**Authors:** Sergej Wüthrich, Caroline Sahli Lozano

**Affiliations:** Institute for Research, Development, and Evaluation, Bern University of Teacher Education, Bern, Switzerland

**Keywords:** inclusive education, attitudes, self-efficacy, pre- and in-service teachers, special education teachers, measurement invariance, psychometric scale development and validation

## Abstract

**Introduction:**

This study examined the psychometric properties of two widely used scales measuring teacher attitudes (Attitudes to Inclusion Scale; AIS) and self-efficacy (Teacher Efficacy for Inclusive Practices Scale; TEIP and its short form TEIP-SF) toward inclusive education.

**Methods:**

Using a sample of Swiss teachers (*N* = 1,546), including pre-service regular teachers (*n* = 147), in-service regular teachers (*n* = 1,168), and special education teachers (*n* = 231), we applied confirmatory factor analysis (CFA) and exploratory structural equation modeling (ESEM) to assess factor structure and assessed reliability, convergent validity and discriminant validity of the scales.

**Results:**

Results indicated that ESEM models outperformed CFA models. While the AIS and TEIP-SF demonstrated strong psychometric properties across all evaluation criteria, the TEIP showed limitations in convergent and discriminant validity, particularly in its collaboration factor. Alignment procedures demonstrated approximate measurement invariance of all scales across teacher groups. Latent mean comparisons revealed that special education teachers reported significantly more positive attitudes (*d* = 0.74–0.88) and higher self-efficacy in instruction (*d* = 0.69–0.72) and collaboration (*d* = 0.74–0.78) compared to in-service regular teachers. Pre-service teachers also showed more positive beliefs (*d* = 0.44) but lower self-efficacy in behavior management (*d* = 0.22–0.25) than in-service teachers.

**Discussion:**

The findings support the use of the AIS and TEIP-SF for assessing attitudes and self-efficacy toward inclusion across different teacher groups and support domain-specificity of teacher self-efficacy.

## 1 Introduction

Inclusive education aims to ensure that all students, regardless of their abilities or disabilities, have equal opportunities to learn and succeed. Demands to include students with special education needs (SEN) in regular schools are increasing internationally (The United Nations, 2006; UNESCO, 1994). In this context, increasing attention is being paid to the role of teachers in creating inclusive learning environments, particularly to their attitudes toward inclusion and their self-efficacy in implementing inclusive practices.

Attitudes and self-efficacy are central in theoretical frameworks such as the theory of planned behavior (Ajzen, 1991) or Bandura’s social cognitive theory (1997). According to the Theory of Planned Behavior (Ajzen, 1991), attitudes are one of the key determinants of behavioral intentions, alongside subjective norms and perceived behavioral control, the latter corresponding to self-efficacy. Social Cognitive Theory (Bandura, 1997) posits that self-efficacy beliefs and attitudes reciprocally influence each other through mastery experiences and social persuasion, and empirical research has found a moderate correlation between teacher attitudes toward inclusion and their self-efficacy for inclusive practices (*r* = 0.35 in the meta-analysis by Yada et al., 2022).

Unsurprisingly, teacher attitudes and self-efficacy in implementing inclusive practices are among the most studied teacher aspects in inclusive education research (Van Mieghem et al., 2018; Yada et al., 2022), highlighting their role in shaping teacher behavior and practice. They are important predictors of teachers’ intention to adapt their teaching to include students with SEN in their classrooms (Hellmich et al., 2019; Opoku et al., 2020; Sharma and Mannan, 2015) and are negatively related to teacher stress regarding inclusive education (Galaterou and Antoniou, 2017; Nagase et al., 2020). Research in this area not only helps to clarify the impact of inclusion attitudes and self-efficacy on classroom practice but also to better prepare teachers for the challenge of inclusive teaching (Avramidis and Norwich, 2002; Sharma and Nuttal, 2016).

### 1.1 Attitudes and self-efficacy regarding inclusive education: teacher group differences

Research on teacher attitudes and self-efficacy toward inclusive education reveals consistent patterns of differences across teacher groups. Regarding attitudes, special education teachers consistently demonstrate more positive attitudes toward inclusion compared to regular teachers, with relatively large and consistent effects documented across many countries (Guillemot et al., 2022). In contrast, comparisons between pre-service and in-service teachers yield more mixed results. While some individual studies suggest that younger teachers or those with less experience hold more favorable attitudes (Avramidis and Norwich, 2002), recent meta-analyses have not found significant overall differences in attitudes between pre-service and in-service teachers (Guillemot et al., 2022; van Steen and Wilson, 2020).

The pattern for self-efficacy is more complex and less well-established, partly due to fewer direct comparative studies. Research generally indicates that specialization and experience are associated with higher self-efficacy (Wray et al., 2022). Special education teachers, with their specialized training, report higher self-efficacy than regular education teachers, particularly in areas such as managing disruptive student behavior (Kazanopoulos et al., 2022). When comparing pre-service and in-service teachers, findings are mixed: while some studies show in-service teachers reporting higher self-efficacy than pre-service teachers (Sokal and Sharma, 2017), others suggest that pre-service teachers may begin with inflated self-efficacy due to idealized views of teaching, which subsequently declines upon entering practice (Pendergast et al., 2011; Weinstein, 1988; Woolfolk Hoy and Spero, 2005).

However, these group differences must be interpreted cautiously, as they may reflect not only genuine differences in attitudes and self-efficacy, but also systematic differences in how these constructs are understood and interpreted by different teacher groups. Pre-service teachers may lack practical referents for inclusive practices, in-service regular teachers may interpret inclusion through their classroom-based experiences, while special education teachers may understand inclusion through their often specialized support roles (e.g., providing individualized one-to-one or small group teaching; Paulsrud and Nilholm, 2020). These varying professional contexts and experiences may influence not only the levels of reported attitudes and self-efficacy, but also the very meaning of these constructs across groups.

### 1.2 Measurement challenges

Differential interpretation of psychological constructs poses significant challenges for measurement and comparison. Pre-service teachers, in-service teachers and special education teachers differ in their levels of education, specialized training, teaching experience, and professional roles. These differences in background and experience may influence how teachers interpret and respond to survey items about inclusion attitudes and self-efficacy, potentially not only affecting their overall levels of attitudes and self-efficacy, but also affecting the way items function as indicators of these constructs.

When items function differently across groups, it is important to consider the possible source of this measurement non-invariance, i.e., whether this differential functioning reflects methodological artifacts or genuine differences in how attitudes and self-efficacy manifest across professional contexts. For instance, special education teachers may evaluate “managing challenging student behaviors” against different standards than pre-service teachers, reflecting their expertise rather than measurement error. Such construct-relevant differences in item interpretation may result in measurement non-invariance. Conversely, differential item functioning in measures of fundamental pedagogical beliefs (e.g., “I believe that all students can learn in inclusive classrooms if their teachers are willing to adapt the curriculum”) would more likely suggest measurement artifacts, as such core beliefs should be conceptually independent of professional experience or specialization.

Despite the importance of assessing measurement invariance for valid group comparisons, it has been rarely evaluated in studies comparing pre-service, in-service, and special education teachers with notable exceptions such as Miesera et al. (2019). Given that these groups differ systematically in training and experience—factors that may influence how certain items are interpreted—employing the alignment method (Asparouhov and Muthén, 2014) to assess approximate measurement invariance and compare latent means might be better suited than traditional multi group confirmatory factor analysis. The alignment approach allows for some degree of measurement non-invariance across groups while optimizing comparability of latent means, and indicates which parameters show the greatest variation across groups. This enables both valid latent mean comparisons and exploratory examination of item-level differences that may reflect genuine contextual variation in how constructs manifest, and addresses a significant gap in the literature, as conclusions about teacher group differences regarding inclusion attitudes and self-efficacy did not consider measurement (non-) invariance so far.

### 1.3 Measurement instruments

In inclusive education contexts, the two constructs of attitudes and self-efficacy are typically assessed using standardized measurement instruments, such as the Attitudes to Inclusion Scale (AIS; Sharma and Jacobs, 2016), and the Teacher Efficacy in Inclusive Practices Scale (TEIP; Sharma et al., 2012). Both AIS and TEIP have been widely used in research on inclusive education, with several studies examining their psychometric properties across different contexts. The AIS, developed by Sharma and Jacobs (2016), was initially tested using in-service teacher samples from India and Australia, where two unidimensional factors (beliefs and feelings about inclusion) were established. Although not explicitly assessed, later studies confirmed the proposed two-factor structure in various contexts using confirmatory factor analyses (CFA). For instance Miesera et al. (2019) confirmed the two-factor structure in two samples of German pre-service regular and special education teachers. They also demonstrated partial scalar measurement invariance of the scale across the two samples, indicating that the found differences of significantly more positive feelings toward inclusion (but not more positive beliefs) among pre-service special education teachers were meaningful. In other studies, the two-factor structure of the AIS and partial scalar invariance could be reliably confirmed across in-service teacher samples from four out of five countries (in the Canadian, German, Italian, and Swiss, but not in the Greek teacher sample; Sahli Lozano et al., 2024a), as well as across two pre-service special education teacher samples from Italy and Switzerland (Sahli Lozano et al., 2024b).

The TEIP (Sharma et al., 2012) was developed based on existing teacher efficacy scales, such as the widely used Teacher Efficacy Scale (Tschannen-Moran and Hoy, 2001), but has been adapted specifically to inclusive education settings. Since its development, it has undergone extensive psychometric evaluation. The original validation study, conducted with pre-service teachers from Canada, Australia, Hong Kong, and India, established a three-factor structure through exploratory factor analysis: efficacy to use inclusive instruction, efficacy in collaboration, and efficacy in managing behavior. Several subsequent studies have examined the TEIP’s psychometric properties. Although not all studies could readily establish the originally proposed factor structure (e.g., Miesera et al., 2019; Park et al., 2016; Sahli Lozano et al., 2023), the TEIP is probably the most frequently used scale to assess teacher efficacy with regard to inclusive education (Sahli Lozano et al., 2023).

Recently, a short form of the TEIP (TEIP-SF) was developed to address earlier concerns regarding the factor structure and to reduce survey length while maintaining good psychometric integrity. The shorter version is particularly valuable for large-scale assessments and studies combining multiple instruments, where participant fatigue and response quality are concerns (Sahli Lozano et al., 2023). This nine-item version (compared to the original 18-item scale) demonstrated favorable psychometric properties using CFA across Swiss, Australian, and Canadian in-service teacher samples. The TEIP-SF retained the three-factor structure of the original scale while showing adequate reliability, factorial validity, and partial scalar measurement invariance across the three samples. These favorable properties were also demonstrated in two further studies (Sahli Lozano et al., 2024a; Sahli Lozano et al., 2024b).

### 1.4 Research gaps and methodological considerations

While these validation studies have provided valuable evidence for the use of the AIS and TEIP, several important gaps remain.

First, assessing the quality of measurement scales thoroughly is essential for valid conclusions, particularly when scales are translated or used across different teacher populations. While many studies rely on Cronbach’s alpha and CFA fit indices, these metrics alone are inadequate because they assume equal factor loadings and good model fit does not guarantee measurement quality (Fornell and Larcker, 1981). Instead, comprehensive assessment should employ multiple criteria for evaluating reliability, convergent validity, and discriminant validity (Cheung et al., 2024).

Second, while most studies have examined single teacher populations, research comparing multiple teacher groups has rarely addressed measurement non-equivalence. This study examines measurement properties across pre-service, in-service, and special education teachers using alignment optimization (Asparouhov and Muthén, 2014), quantifying the degree to which items function differently across groups while enabling latent mean comparisons. This addresses whether differences in training and experience affect not only teachers’ levels of attitudes and self-efficacy, but also how these constructs are measured.

Third, previous validation studies have relied predominantly on traditional CFA approaches. While CFA is widely used in scale validation, its strict requirement of zero cross-loadings can lead to artificially inflated factor correlations and potentially misspecified factor structures (Asparouhov and Muthén, 2009; Morin et al., 2013). This limitation may be particularly relevant for constructs like attitudes and self-efficacy, where some degree of overlap between statements or factors might be difficult to avoid and theoretically meaningful. Exploratory structural equation modeling (ESEM) represents a connection between EFA (exploratory factor analysis) measurement models and the overarching CFA framework, making it possible to benefit from all of the advantages typically associated with CFA, while relying on an EFA measurement model by incorporating cross-loadings (Asparouhov and Muthén, 2009). The development of target rotation allows for the specification of the main factor loadings, while constraining the cross-loadings to be as close to zero as possible, yet allowing them to be freely estimated (Marsh et al., 2014). To date and to our knowledge, no studies have employed ESEM to examine the factor structure of the AIS, the TEIP, or the TEIP-SF, which might resolve earlier reported issues (e.g., regarding the full-length TEIP, Sahli Lozano et al., 2023).

By using ESEM in combination with rigorous tests of validity and invariance, the present study aims to contribute both methodological advancements and theoretical clarity regarding the structure and comparability of key constructs in inclusive education.

### 1.5 The present study

Building on these goals, the study comprises a comprehensive psychometric evaluation of the AIS, TEIP, and TEIP-SF in a large sample of Swiss teachers, including pre-service regular teachers (PSRT), in-service regular teachers (ISRT), and special education teachers (SET). Specifically, the study pursues four objectives:

First, to examine and compare the factor structures of the AIS, TEIP, and TEIP-SF using both CFA and ESEM approaches.

Second, to assess the reliability, convergent validity, and discriminant validity of the three instruments.

Third, to investigate measurement invariance across the three teacher groups to ensure valid group comparisons.

And fourth, to compare attitudes and self-efficacy levels across teacher groups, contingent on establishing measurement invariance.

By addressing these objectives, this study will investigate the evidence for the valid use of these instruments in comparing different teacher groups, offer insights into potential measurement challenges, and provide suggestions in how to further improve these scales to assess attitudes and self-efficacy toward inclusive education across different professional teacher populations.

## 2 Methods

### 2.1 Participants

Participants were invited to fill in an online survey and were recruited in three ways. First, teachers were contacted by a collaboration with a regional professional association of teachers, which sent the link to the survey to their members. Second, the link to the survey to school principals of 200 randomly selected schools in the German-speaking part of Switzerland, with the request to forward it to their teachers. Third, the link to the survey was sent to pre-service teacher students at the Bern University of Teacher Education. A total of 2009 participants filled in the survey. Of these, the following individuals were excluded: (a) individuals currently not working as regular or special teachers and not registered as pre-service teachers at pre-primary, primary or lower secondary school levels, (b) special education teacher students and (c) special education teachers working in special schools or classes. The rationale for these exclusion criteria were to reduce heterogeneity within groups as best as possible. A total of 1,546 pre- and in-service regular teachers and special education teachers matched these criteria. Due to anonymous data collection, an accurate response rate cannot be reported. However, regarding teaching level, sex, and age, the sample characteristics are comparable to the general teacher population in Switzerland (Swiss Federal Statistical Office, 2018). Descriptive statistics for the three teacher samples (PSRT, ISRT, and SET) is displayed in Table 1.

**Table 1:** Sample characteristics.

Variable	Total		ISRT		PSRT		SET	
	*n*	%	*n*	*%*	*n*	*%*	*n*	*%*
Total	1,546	100	1,168	75.5	147	9.5	231	14.9
Age								
<25 years	161	10.4	55	4.7	106	72.1	0	0
25–30 years	203	13.1	172	14.7	22	15.0	9	3.9
31–40 years	274	17.7	218	18.7	11	7.5	45	19.5
>40 years	908	58.7	723	61.9	8	5.4	177	76.6
Sex								
Female	1,220	79.7	908	78.4	113	77.9	199	87.7
Male	310	20.3	250	21.6	32	22.1	28	12.3
Teaching experience								
<1 year	123	8.0	19	1.6	104	70.7	0	0
1–3 years	144	9.3	107	9.2	33	22.4	4	1.7
4–10 years	252	16.3	217	18.6	8	5.4	27	11.7
>10 years	1,027	66.4	825	70.6	2	1.4	200	86.6
Teaching level								
Preschool/primary	1,115	72.1	894	76.5	29	19.7	192	83.1
Secondary	326	21.1	274	23.5	13	9.8	39	16.9
Other	105	6.8	0	0	105	71.4	0	0

ISRT, in-service regular school teachers; PSRT, pre-service regular school teachers; SET, special education teachers.

### 2.2 Instruments

The German adaption of the AIS and TEIP by Gebhardt et al. (2018) was employed for this study, as well as the TEIP-SF version from Sahli Lozano et al. (2023). Additionally, sociodemographic as well as profession-related background information from the participants (gender, age, teaching experience in years, and teaching level) were collected.

#### 2.2.1 Attitudes to Inclusion Scale (AIS)

The Attitudes to Inclusion Scale (AIS), developed by Sharma and Jacobs (2016), assesses teachers’ attitudes toward inclusive education. It comprises eight items. Example items are “I believe that all students, regardless of their ability, should be taught in regular classrooms” and “I am excited to teach students with a range of abilities in my class.” Participants indicate their agreement on a 7-point Likert-type scale ranging from strongly disagree (1) to strongly agree (7). The first subscale (items 1–4) is intended to measure beliefs about inclusion, while the second subscale is intended to measure teachers’ feelings about inclusion (items 5–8).

#### 2.2.2 Teacher Efficacy in Inclusive Practices (TEIP and TEIP-SF)

The Teachers’ Efficacy in Inclusive Practices (TEIP) Scale by Sharma et al. (2012) assesses teachers’ self-efficacy beliefs in inclusive teaching. It comprises 18 items. Example items are “I am able to provide an alternate explanation, for example, when students are confused” and “I am able to work jointly with other professionals and staff (e.g., aides, other teachers) to teach students with disabilities in the classroom.” Participants indicate their agreement on a 6-point Likert-type scale ranging from strongly disagree (1) to strongly agree (6). The three sub-factors each consist of six items: Efficacy to use inclusive instructions, efficacy in managing behavior, and efficacy in collaboration. In addition to the TEIP, the short form TEIP-SF was also developed. Instead of 18 items, the TEIP-SF uses only nine items in total (three items per sub factor).

### 2.3 Statistical analysis

All analyses were performed using M plus Version 8.11 (Muthén and Muthén, 2021; R Core Team, 2024). The analyses included the following steps: Step 1: Performing confirmatory factor analysis and exploratory structural equation modeling (ESEM) on the AIS, the TEIP, and the TEIP-SF scales, using three variants: a one-factor CFA model, a two-factor (AIS) or three-factor (TEIP and TEIP-SF) CFA model, and the corresponding ESEM model. Subsequently, the best fitting model for each scale was selected for further analyses, which included: Step 2: evaluating reliability, convergent validity, and discriminant validity; Step 3: comparing latent means of attitudes and self-efficacy components across the three groups.

#### 2.3.1 Confirmatory factor analyses and exploratory structural equation modeling

For all analyses of the three scales with three models each, model parameters were estimated using the maximum likelihood estimator with robust standard errors (MLR) to account for non-normal data distribution. Model fit was evaluated using multiple fit indices: the comparative fit index (CFI), the root mean square error of approximation (RMSEA), and the standardized root mean square residual (SRMR). CFI > 0.90, RMSEA <0.08 and SRMR <0.08 were considered minimal acceptable fit indices (Hu and Bentler, 1999). The best fitting model was evaluated by comparing models with acceptable fit using multiple criteria: the Satorra-Bentler scaled chi-square difference test and changes in approximate fit indices, where ΔCFI ≤ 0.010 (decrease), ΔRMSEA ≤ 0.015 (increase), and ΔSRMR ≤0.010 (increase) indicated equivalent fit (Chen, 2007). Additionally, information criteria (AIC, BIC, and sample-size adjusted BIC) were examined, with lower values indicating better fit, and factor correlations were considered (where lower correlations in ESEM might be indicative of inflated factor correlations in CFA; Van Zyl and Ten Klooster, 2022).

#### 2.3.2 Reliability, convergent and discriminant validity

Reliability determines the maximum possible validity of scales and affects the accuracy of group comparisons. Convergent validity demonstrates that items assumed to measure the same construct are sufficiently related, while discriminant validity ensures that theoretically distinct constructs (e.g., beliefs vs. feelings about inclusion) are empirically distinguishable. The approaches presented by Rönkkö and Cho (2022) were followed. Scale reliability was assessed by calculating composite reliability (CR), using 0.7 as the minimum acceptable threshold (Hair et al., 2019). For convergent validity, two criteria were used: standardized factor loadings, using >0.5 as an indicator of sufficient factor loading (Hair et al., 2019), and the Average Variance Extracted (AVE) calculated for each factor, with values above 0.5 being adequate (Fornell and Larcker, 1981). Discriminant validity was evaluated using three criteria: examining item cross-loadings, where absence of loadings above 0.3 on non-target factors was used as a criterion; comparing the AVE with shared variance (where the AVE should be larger than the squared correlations with other factors; AVE-SV approach; Fornell and Larcker, 1981); and examining factor correlations, where values below 0.8 indicated discriminant validity (Rönkkö and Cho, 2022).

#### 2.3.3 Alignment and latent mean comparisons

For the best fitting models, measurement invariance across the three teacher groups was assessed using the alignment method (Asparouhov and Muthén, 2014, 2023). In the alignment approach, a configure model in which factor loadings and intercepts are freely estimated across groups is estimated, and an optimal rotation that minimizes the total amount of measurement non-invariance is then identified. This method allows for approximate rather than strict measurement invariance, making it appropriate when some degree of parameter variation across groups is expected but overall comparability of latent constructs is maintained. Following established guidelines (Asparouhov and Muthén, 2014), the degree of measurement invariance was evaluated by examining the proportion of non-invariant parameters. Approximate measurement invariance was considered supported if fewer than 25% of parameters showed substantial non-invariance. All alignment analyses were conducted using maximum likelihood estimation with robust standard errors (MLR) and the free alignment procedure was used, unless unreliable estimates of standard errors required the use of fixed alignment (Asparouhov and Muthén, 2014). After approximate measurement invariance was established, latent factor means were compared across teacher groups, with in-service regular teachers used as the reference group. Effect sizes were calculated using latent variable Cohen’s *d* (Hancock, 2001), where values of 0.2, 0.5, and 0.8 represent small, medium, and large effects, respectively (Cohen, 1988).

## 3 Results

### 3.1 Descriptive results

Raw (manifest) mean scores for the different samples and scales are shown in Table 2.

**Table 2:** Raw mean scores of AIS, TEIP and TEIP-SF across groups/correlations among scales and sample characteristics.

Scale	Variable	Composite scores *M (SD)*			Correlations										
		ISRT	PSRT	SET	BEL	INS	MB	COL	INS_SF_	MB_SF_	COL_SF_	Sex	Age	Tex	Tlv
AIS	FEEL	4.06 (1.68)	4.80 (1.45)	5.27 (1.48)	**0.72**	**0.33**	**0.18**	**0.49**	**0.38**	**0.16**	**0.48**	**0.13**	**−0.12**	**−0.09**	**−0.11**
	BEL	4.53 (1.51)	5.02 (1.19)	5.80 (1.18)		**0.41**	**0.25**	**0.53**	**0.45**	**0.23**	**0.52**	**0.18**	**−0.11**	**−0.08**	**−0.18**
TEIP	INS	4.70 (0.75)	4.57 (0.70)	5.12 (0.64)			**0.51**	**0.61**	**0.90**	**0.50**	**0.55**	**0.09**	**0.06**	**0.10**	**−0.06**
	MB	4.65 (0.86)	4.46 (0.85)	4.70 (0.81)				**0.47**	**0.38**	**0.94**	**0.37**	**−0.08**	0.03	**0.08**	0.04
	COL	4.31 (0.86)	4.33 (0.77)	4.95 (0.77)					**0.60**	**0.45**	**0.92**	**0.14**	**0.07**	**0.10**	**−0.11**
TEIP-SF	INS_SF_	4.51 (0.95)	4.53 (0.77)	5.14 (0.77)						**0.37**	**0.56**	**0.14**	0.01	0.05	**−0.08**
	MB_SF_	4.77 (0.89)	4.56 (0.88)	4.84 (0.82)							**0.35**	−0.03	0.04	**0.09**	0.02
	COL_SF_	4.38 (1.09)	4.43 (0.91)	5.10 (0.85)								**0.16**	0.04	**0.06**	**−0.14**
	Sex												**−0.10**	**−0.07**	**−0.42**
	Age													**0.83**	**0.06**
	Tex														0.05

ISRT, in service regular school teachers; PSRT, preservice regular school teachers; SET, special education teachers. AIS, attitudes toward inclusion scale (FEEL, Feelings; BEL, Beliefs); TEIP, teacher efficacy for inclusive practices scale (INS, instruction, MB, managing behavior, COL, collaboration); TEIP-SF, teacher efficacy for inclusive practice scale–short form; Tex, teaching experience; Tlv, teaching level. Correlations were calculated based on the entire sample. Pearson correlation was used for metric variables and spearman rank for ordered variables. Significant correlations (*p* < 0.05) are printed in bold.

### 3.2 CFA and ESEM models for AIS, TEIP and TEIP-SF

Table 3 summarizes the results of the one-factor CFA model, a multi-factor CFA model (representing the originally proposed factor structure), and the corresponding multi-factor ESEM model.

**Table 3:** Model comparisons for AIS, TEIP, and TEIP-SF.

Sample	Model	Type	*χ* ^2^	df	CFI	RMSEA	SRMR	AIC	BIC	aBIC	Meets criteria
AIS	Model 1	1-Factor CFA	529.511	20	0.906	0.129	0.050	43,369	43,497	43,421	No
AIS	Model 2	2-Factor CFA	130.999	19	0.979	0.062	0.026	42,830	42,963	42,884	Yes
**AIS**	**Model 3**	**2-Factor ESEM**	**78.157**	**13**	**0.988**	**0.057**	**0.015**	**42,769**	**42,934**	**42,836**	**Yes**
TEIP	Model 1	1-Factor CFA	3,128.944	136	0.664	0.124	0.183	65,799	66,077	65,909	No
TEIP	Model 2	3-Factor CFA	1,276.730	132	0.872	0.078	0.086	62,831	63,131	62,950	No
**TEIP**	**Model 3**	**3-Factor ESEM**	**627.613**	**102**	**0.941**	**0.060**	**0.030**	**61,874**	**62,332**	**62,055**	**Yes**
TEIP-SF	Model 1	1-Factor CFA	1,662.967	28	0.599	0.203	0.189	34,195	34,331	34,249	No
TEIP-SF	Model 2	3-Factor CFA	91.567	24	0.983	0.045	0.027	31,850	32,008	31,913	Yes
**TEIP-SF**	**Model 3**	**3-Factor ESEM**	**29.348**	**12**	**0.996**	**0.032**	**0.008**	**31,784**	**32,005**	**31,872**	**Yes**

Bold text indicates the best-fitting model for each scale. *χ*^2^, chi-square test statistic; df, degrees of freedom; CFI, comparative fit index; RMSEA, root mean square error of approximation; SRMR, standardized root mean square residual; AIC, Akaike information criterion; BIC, Bayesian information criterion; aBIC, sample size adjusted BIC. Model fit criteria: CFI ≥ 0.95, RMSEA ≤0.06, SRMR ≤0.08.

For the AIS, both the two-factor CFA (*χ*^2^ = 130.999, df = 19, CFI = 0.979, RMSEA = 0.062, SRMR = 0.026) and two-factor ESEM model (*χ*^2^ = 78.157, df = 13, CFI = 0.988, RMSEA = 0.057, SRMR = 0.015) showed good fit to the data. Model comparison results [Satorra-Bentler corrected Δ*χ*^2^ (6) = 74.47, *p* < 0.001; ΔCFI = 0.009, ΔRMSEA = 0.005, ΔSRMR = 0.011] indicated a marginally better fit for the ESEM model, with factor correlations being slightly lower in the ESEM model (0.80 vs. 0.82). Therefore, the ESEM model was retained.

For the TEIP, while neither CFA model showed acceptable fit, the three-factor ESEM model demonstrated good fit to the data (*χ*^2^ = 627.613, df = 102, CFI = 0.941, RMSEA = 0.060, SRMR = 0.030). Hence, the ESEM model was retained.

For the TEIP-SF, both the three-factor CFA (*χ*^2^ = 91.567, df = 24, CFI = 0.983, RMSEA = 0.045, SRMR = 0.027) and the ESEM model (*χ*^2^ = 29.348, df = 12, CFI = 0.996, RMSEA = 0.032, SRMR = 0.008) showed good fit. Model comparison results [Δ*χ*^2^ (12) = 93.78, *p* < 0.001; ΔCFI = 0.013, ΔRMSEA = 0.013, ΔSRMR = 0.019] indicated better fit for the ESEM model, with factor correlations being slightly lower in the ESEM model (Δ*r* = 0.006 to Δ*r* = 0.022). Therefore, the ESEM model was retained.

### 3.3 Reliability, convergent and discriminant validity

Scale reliability, convergent validity, and discriminant validity were examined for all factors across the three scales based on the ESEM model (see Table 4).

**Table 4:** Reliability, convergent and discriminant validity measures for the ESEM models.

Scale/factor	CR	Min TL	AVE	Max CL	Max *r*	AVE-SV	REL	CV	DV
AIS/BEL	0.875	0.660	0.640	0.219	0.795	Yes	Yes	Yes	Yes
AIS/FEEL	0.851	0.543	0.596	0.120	0.795	**No**	Yes	Yes	**No**
TEIP/INS	0.803	**0.476**	**0.413**	0.297	0.672	**No**	Yes	**No**	**No**
TEIP/MB	0.891	0.539	0.587	0.295	0.579	Yes	Yes	Yes	Yes
TEIP/COL	0.764	**0.204**	**0.397**	0.218	0.672	**No**	Yes	**No**	**No**
TEIP-SF/INS	0.809	0.663	0.590	0.098	0.693	Yes	Yes	Yes	Yes
TEIP-SF/MB	0.883	0.763	0.718	0.049	0.457	Yes	Yes	Yes	Yes
TEIP-SF/COL	0.840	0.739	0.639	0.130	0.693	Yes	Yes	Yes	Yes

CR, composite reliability; Min TL, minimum target loading; AVE, average variance extracted; Max CL, maximum cross-loading; Max *r*, maximum factor correlation; FLC, Fornell-Larcker criterion; REL, reliability; CV, convergent validity; DV, discriminant validity. Criteria for acceptable values: CR > 0.8 for reliability; Min TL > 0.5 and AVE > 0.5 for convergent validity; Max CL <0.3, Max R <0.85, and AVE-SV (AVE > shared variance) for discriminant validity. Indices that did no meet criterion are printed in bold.

For the AIS scale, both factors showed good reliability (CR > 0.70) with values of 0.875 for beliefs and 0.851 for feelings. The beliefs factor demonstrated good convergent validity with all target loadings above 0.50 (minimum = 0.660) and adequate AVE (0.640). While the feelings factor also showed acceptable target loadings (minimum = 0.543) and AVE (0.596), it did not meet the AVE-SV criterion for discriminant validity mainly because of the high factor correlation (0.795), which also approached the 0.80 threshold.

For the TEIP scale, the managing behavior factor showed strong psychometric properties across all criteria (CR = 0.891, minimum target loading = 0.539, AVE = 0.587). However, both instruction and collaboration factors showed limitations in convergent validity (with some target loadings and AVE clearly below 0.50) and discriminant validity (AVE-SV criterion not met). Especially two items of the collaboration factor demonstrated low standardized loadings on the target factor (item 13 and item 14 with 0.252 and 0.204, respectively).

The TEIP-SF demonstrated the strongest psychometric properties, with all three factors showing good reliability (CR ranging from 0.809 to 0.883), convergent validity (all target loadings > 0.66 and AVE > 0.59), and discriminant validity (cross-loadings <0.13, factor correlations <0.70, and all factors meeting the AVE-SV criterion).

### 3.4 Approximate measurement invariance assessment

The free alignment procedure yielded in all cases unreliable estimates of standard error, so the fixed alignment procedure was specified with latent means of the group of ISRT fixed to zero. For the AIS and the TEIP scale, aligned ESEM (AESEM) models were used. However, the AESEM model failed to converge for the TEIP-SF scale due to estimation problems in the smallest group of PRST, including a negative residual variance. Therefore, it was necessary to fall back to an aligned CFA model instead.

For all three scales, approximate measurement invariance was supported. For the AIS scale, one of 8 intercepts (12.5%) showed non-invariance (item 7: “I am pleased that including students with a range of abilities will make me a better teacher”), where the group of PSRT (4.761) differed significantly from both ISRT (4.113, *p* < 0.001) and SET (3.873, *p* < 0.001), while all 16 loadings across both factors showed invariance across the three groups. For the TEIP scale, all 18 intercepts showed invariance across groups, while one of 54 loadings (1.9%) showed non-invariance in its factor loading (item 13: “I am confident in my ability to get parents involved in school activities of their children with disabilities”), where the group of ISRT (0.166) differed significantly from PSRT (0.679, *p* = 0.011) and SET (0.685, *p* = 0.002). Finally, for the TEIP-SF scale, one of 9 intercepts (11.1%) showed non-invariance (item 3: “I am confident in designing learning tasks so that the individual needs of students with disabilities are accommodated”), where pre-service teachers (3.951) differed significantly from both in-service regular teachers (4.320, *p* < 0.001) and special education teachers (4.527, *p* < 0.001), while all 9 loadings showed invariance across the three groups.

### 3.5 Latent mean differences

Because all models showed a sufficiently low number of non-invariant factor loadings and intercepts (all <25%), approximate measurement invariance holds and estimated factor mean comparisons indicate valid group differences. Results can be found in Table 5.

**Table 5:** Latent mean differences across groups.

Scale	PSRT (vs. ISRT)	Effect size (*d*)	SET (vs. ISRT)	Effect size (*d*)
AIS: BEL	0.441^**^	0.44	0.735^***^	0.74
AIS: FEEL	0.187	0.19	0.875^***^	0.88
TEIP: INS	−0.302	0.30	0.690^***^	0.69
TEIP: MB	−0.217^*^	0.22	−0.034	0.03
TEIP: COL	0.129	0.13	0.781^***^	0.78
TEIP-SF: INS	0.152	0.16	0.702^***^	0.72
TEIP-SF: MB	−0.248^*^	0.25	0.032	0.03
TEIP-SF: COL	0.124	0.13	0.712^***^	0.74

*d*, Cohen’s d (effect size). ^*^p < 0.05, ^***^p < 0.001.

For the AIS scale, both PRST (*M* = 0.441, *SE* = 0.148, *p* = 0.003, *d* = 0.44) and SET (*M* = 0.735, *SE* = 0.079, *p* < 0.001, *d* = 0.74) showed significantly more positive beliefs about inclusion than IRST. For feelings about inclusion, SET (*M* = 0.875, *SE* = 0.206, *p* < 0.001, *d* = 0.88) showed significantly more positive feelings than ISRT, while PSRT (*M* = 0.187, *SE* = 0.097, *p* = 0.055, *d* = 0.19) did not differ from ISRT.

For the TEIP scale, SET (*M* = 0.690, *SE* = 0.147, *p* < 0.001, *d* = 0.69) had significantly higher efficacy beliefs regarding instructions than ISRT, while PSRT (*M* = −0.302, *SE* = 0.366, *p* = 0.410, *d* = 0.30) did not differ from ISRT. For managing behavior, PRST (*M* = −0.217, *SE* = 0.097, *p* = 0.025, *d* = 0.22) had significantly lower efficacy beliefs than ISRT, while SET (*M* = −0.034, *SE* = 0.087, *p* = 0.695, *d* = 0.03) did not differ from ISRT. For collaboration, SET (*M* = 0.781, *SE* = 0.164, *p* < 0.001, *d* = 0.78) had significantly higher efficacy beliefs than ISRT, while PSRT (*M* = 0.129, *SE* = 0.168, *p* = 0.441, *d* = 0.13) did not differ from ISRT.

For the TEIP-SF, patterns were similar to the full TEIP with SET (*M* = 0.702, *SE* = 0.103, *p* < 0.001, *d* = 0.70) having significantly higher efficacy beliefs regarding instructions than ISRT, while pre-service teachers (*M* = 0.152, *SE* = 0.095, *p* = 0.109, *d* = 0.15) did not differ from ISRT. For managing behavior, PRST (*M* = −0.248, *SE* = 0.096, *p* = 0.010, *d* = 0.25) had significantly lower efficacy beliefs than ISRT, while SET (*M* = 0.032, *SE* = 0.074, *p* = 0.664, *d* = 0.03) did not differ from ISRT. For collaboration, SET (*M* = 0.712, *SE* = 0.067, *p* < 0.001, *d* = 0.71) had significantly higher efficacy beliefs than ISRT, while PSRT (*M* = 0.124, *SE* = 0.090, *p* = 0.166, *d* = 0.12) did not differ from ISRT.

## 4 Discussion

The AIS and TEIP scales are frequently used measures to assess teacher attitudes and self-efficacy toward inclusive education (Miesera et al., 2019; Sahli Lozano et al., 2023). Here, we thoroughly assessed these scales (including the short form of the TEIP) regarding their psychometric properties and factorial structures, applying both CFA and ESEM approaches, in a large sample of Swiss teachers. We assessed whether the scales can be validly employed across teacher groups differing in knowledge, teaching experience, and specialization (i.e., pre-service regular teachers, in-service regular teachers, and special education teachers), and whether approximate measurement invariance can be established across groups to meaningfully compare group differences in attitudes and self-efficacy between through latent mean comparisons.

### 4.1 Factor structure and measurement quality of the AIS, TEIP, and TEIP-SF

Our study provides a comprehensive evaluation of the psychometric properties of the AIS, TEIP, and TEIP-SF using both traditional CFA and more flexible ESEM approaches. Although the multi-factor CFA models for the AIS and TEIP-SF provided adequate fit, for all three scales, the ESEM models generally outperformed the CFA models. The better fit of ESEM models suggests that too restrictive CFA models with cross-loadings constrained to zero can lead to problems for complex psychological constructs such as inclusion attitudes and self-efficacy (Marsh et al., 2014; Morin et al., 2013). This has been evident in the full-length TEIP, which might explain problems in replicating its original factor structure in some previous studies using CFA models (Miesera et al., 2019; Park et al., 2016; Sahli Lozano et al., 2023). However, even with adequate fit of more restrictive CFA models and small cross-loadings, the latter can lead to inflated parameter estimates and biased results, including inflated correlation of latent factors (Marsh et al., 2014; Van Zyl and Ten Klooster, 2022).

For the AIS, our analysis confirms its originally proposed two-factor structure well. Both beliefs and feelings factors demonstrated good reliability (CR > 0.85) and convergent validity (AVE > 0.59). However, their high correlation (0.795) raises slight concerns regarding discriminant validity, although the better fit of the two-factor model over a single-factor solution indicates that maintaining this theoretical distinction remains valuable, particularly for understanding how different aspects of attitudes might respond to intervention.

The TEIP, despite showing acceptable model fit with a three-factor ESEM, demonstrated some limitations that warrant attention. While the managing behavior factor demonstrated strong psychometric properties, both instruction and collaboration factors showed concerning convergent and discriminant validity issues. Particularly problematic seemed to be the collaboration factor, where two items with standardized loadings below 0.30 were identified. Inspecting the content of the two items revealed that at least one item (item 14: “I can improve the learning of a student who is failing”) did not, or at least not directly, capture collaborative efforts.

Issues with the factor structure of the TEIP have been often reported in the past. Sahli Lozano et al. (2023) reported that out of 16 studies using the TEIP and specifying a CFA model, 11 removed specific items due to factor cross-loadings and to improve model fit. These studies involved a wide range of teacher samples from countries (e.g., Australia, China, Ghana, Finland, Japan, Saudi Arabia, Spain), and although some items seemed more problematic across contexts, item misfit also varied across contexts. Similar observations were made in a scoping review on the psychometric properties of the TEIP (Selenius and Ginner Hau, 2024). Studies using Rasch analyses also questioned the multidimensionality of the TEIP, as they did not find support for the unidimensionality of sub factors (Alnahdi, 2019, using a Saudi Arabian teacher sample), with the exception of the collaboration factor (Alnahdi and Yada, 2020, using a Japanese teacher sample). These findings align with some problematic observations in this study that self-efficacy in instruction and self-efficacy in collaboration of the TEIP do not seem to be clear and distinguishable sub factors in the full-length TEIP. While this finding might be specific to the Swiss context, considering these repeatedly mentioned issues, we propose to be careful and inspect the factor structure when applying the full-length TEIP and interpreting sub factor scores to ensure convergent factor validity.

In contrast, the TEIP-SF emerged as psychometrically sound across all evaluation criteria. With good reliability (CR > 0.80), convergent validity (all target loadings > 0.66), and good discriminant validity (factor correlations <0.70), the TEIP-SF seems to effectively capture distinct aspects of teacher self-efficacy in inclusive settings while maintaining measurement precision. Furthermore, scores from the reduced-length TEIP-SF correspond closely to the full-length scores of the TEIP: latent score correlations range from 0.956 (instruction) to 0.985 (managing behavior). These psychometric properties, combined with its reduced length, suggest it to be a practical and reliable instrument, at least for the Swiss educational context. However, its reduced length also poses greater risks regarding factor reliability when applied to samples in different contexts or when using translated versions (see also Sahli Lozano et al., 2023).

### 4.2 Approximate measurement invariance across teacher groups

The alignment analyses revealed minimal measurement non-invariance across the three teacher groups of PSRT, ISRT and SET, supporting the validity of latent mean comparisons. Only three items showed differential functioning, the intercept of item 7 of the AIS scale, the factor loading of item 13 in the TEIP scale, and the intercept of item 3 in the TEIP-SF scale. These patterns might indicate meaningful contextual differences in how teachers with differing experiences and specializations interpret specific items. The significantly different intercept of item 7 of the AIS scale (“I am pleased that including students with a range of abilities will make me a better teacher”) in the group of PSRT might reflect a more idealistic perspective, and the item’s future orientation and focus on professional growth might also carries different meanings across career stages. Also, ISRT showed significantly lower loadings on the TEIP collaboration factor of item 13 (“I am confident in my ability to get parents involved in school activities of their children with disabilities”) than the other groups, suggesting that they might conceptualize the item in different ways, for example in terms of a classroom management strategy, rather than as professional collaboration. Finally, PRST showed significantly lower intercepts on item 3 of the TEIP-SF (“I am confident in designing learning tasks so that the individual needs of students with disabilities are accommodated”), probably reflecting a lack of practical experience with differentiation. Interestingly, this item showed intercept non-invariance in the TEIP-SF but not in the full TEIP. The ESEM model allowed for cross-loadings that may have absorbed group-specific variance, while the TEIP-SF, due to the small sample of PRST, could not be adequately fitted using AESEM and required the use of the more restrictive CFA model.

### 4.3 Differences in attitudes and self-efficacy of the different teacher groups

Our findings revealed significant differences in attitudes and self-efficacy across Swiss pre-service, in-service, and special education teacher groups that both confirm and extend previous research. The more positive attitudes toward inclusion among special education teachers (with large effects) align with previous meta-analytic results (Guillemot et al., 2022). This underscores the importance of professional development in inclusive education for all teachers. The enhancement of knowledge and competencies pertaining to inclusive practices, both during the initial teacher education program and through ongoing collaboration within multi-professional school teams, might facilitate the mitigation of attitudinal discrepancies. However, to our knowledge, this is the first study directly comparing these different teacher groups using a latent variable approach while ensuring approximate measurement invariance among instruments and teacher groups. These differences likely reflect multiple factors: self-selection effects, with individuals more positively disposed toward inclusion choosing special education careers (Sahli Lozano et al. 2024a), and the impact of specialized training (Sharma and Nuttal, 2016). Interestingly, pre-service teachers also held more positive beliefs toward inclusion than in-service teachers, which might reflect better education or the more idealistic views often held by pre-service teachers (Pendergast et al., 2011). However, these effects were smaller and might be more strongly influenced by country-specific context, as recent meta-analyses could not clearly establish significant differences between pre-service and in-service teacher attitudes internationally (Guillemot et al., 2022; van Steen and Wilson, 2020). Switzerland has only recently moved toward a more inclusive education system over the last two decades. In-service teachers may have received limited or no training in inclusive education during their initial teacher education. Their professional experiences may also have been shaped in more segregated school settings, impacting their beliefs and feelings toward inclusive contexts.

The self-efficacy patterns revealed more complex differences across groups. With both TEIP and TEIP-SF, pre-service teachers demonstrated lower self-efficacy in behavior management than in-service teachers. In contrast, pre-service teachers, despite their lower experience, did not demonstrate significant differences in self-efficacy in instruction and collaboration. While this aligns with research on the theory-practice gap in teacher education (Weinstein, 1988; Woolfolk Hoy and Spero, 2005), this might not be true for self-efficacy in behavior management, which could more strongly align with actual teaching experience. However, the size of the pre-service teacher group was relatively small to detect more subtle differences, so these null findings should be interpreted with caution.

In contrast, patterns for special education teachers were clear-cut: they displayed higher self-efficacy in instruction and collaboration, but similar levels in behavior management compared to in-service regular teachers. While these heightened self-efficacy beliefs reflect the specialized nature of their training and role, aligning with previous research showing generally higher self-efficacy among special educators (Wray et al., 2022), self-efficacy in behavior management seems to be a different factor and less malleable through special education training. Interestingly, previous studies have shown that self-efficacy in behavior management, compared to instruction and collaboration, is the least important predictor of inclusive education practices across teacher samples in different countries (Sahli Lozano et al., 2024b).

## 5 Limitations

Several limitations should be considered when interpreting our findings. First, while our total sample was relatively large, the groups of pre-service (*n* = 147) and special education teachers (*n* = 231) were considerably smaller than the in-service teacher group (*n* = 1,168). This imbalance might have affected the power to detect measurement non-invariance and group differences, and also led to convergence problems when using an AESEM model with the TEIP-SF. Second, due to sample size constraints, we excluded special education teachers working in special schools or classes. Therefore, our findings regarding measurement invariance and group differences apply only to special education teachers working in regular school settings, who might differ from those in segregated settings. Third, psychometric properties and factorial validity of the AIS, TEIP, and TEIP-SF scales were all assessed using German translations of the original scales and a sample of Swiss teachers. Therefore, the findings and scale properties may not generalize to different teacher samples from different linguistic and educational contexts with different inclusive education policies and practices. Fourth, our validation approach focused primarily on internal structure validity, examining factor structure, reliability, and convergent and discriminant validity within the scales themselves. While this provides essential evidence for measurement quality, our evaluation lacks external validity evidence such as correlations with other established measures of related constructs, criterion validity examining how well the scales predict relevant educational outcomes, or convergent validity with alternative measures of teacher attitudes and self-efficacy. Fifth, the cross-sectional nature of our data prevents causal interpretations of group differences. Longitudinal research tracking teachers from pre-service through early career would better illuminate how attitudes and self-efficacy develop and how understandings and concepts regarding inclusive education may or may not change over time.

## 6 Conclusion

Our findings have important implications for future scale development and validation studies in inclusive education research as well as practical implications. First, they demonstrate the value of employing multiple criteria beyond traditional CFA fit indices when evaluating measurement instruments. Second, they demonstrate the necessity that even well-established scales such as the TEIP should be reassessed when using translations or applying the scale to novel teacher samples. Third, they highlight the importance of considering more flexible approaches like ESEM in addition to CFA when assessing scale factor structures. Fourth, they support the use of the AIS and TEIP-SF scales to reliably and validly assess pre-service, in-service and special education teachers’ attitudes and self-efficacy toward inclusion and allow for cross-group comparisons, at least in Swiss teacher samples. Fifth, demonstration of clear discriminant validity among sub factors in the TEIP-SF, together with differential effects in cross-group comparisons, supports the fact that self-efficacy is domain-specific (Bandura, 1997) and has differential effects regarding inclusive education (Sahli Lozano et al., 2024a). Finally, the consistently more positive attitudes and higher self-efficacy among special education teachers indicate the value of teacher training and specialization regarding inclusive education.

## Data Availability

The datasets presented in this study can be found in online repositories. The names of the repository/repositories and accession number(s) can be found at: Daten von Berner und Deutschschweizer Lehrpersonen und sonderpädagogischen Fachpersonen zu Einstellungen, Bedenken und Selbstwirksamkeit gegenüber schulischer Inklusion (ISASI) (2019-2022); https://doi.org/10.48573/7zdp-nq67.
